# Ionic Crosslinking of Linear Polyethyleneimine Hydrogels with Tripolyphosphate

**DOI:** 10.3390/gels10120790

**Published:** 2024-12-03

**Authors:** Luis M. Araque, Antonia Infantes-Molina, Enrique Rodríguez-Castellón, Yamila Garro-Linck, Belén Franzoni, Claudio J. Pérez, Guillermo J. Copello, Juan M. Lázaro-Martínez

**Affiliations:** 1 Universidad de Buenos Aires, Facultad de Farmacia y Bioquímica, Departamento de Ciencias Químicas, Ciudad Autónoma de Buenos Aires 1113, Argentina; lmaraque@conicet.gov.ar (L.M.A.); gcopello@ffyb.uba.ar (G.J.C.); 2 CONICET – Universidad de Buenos Aires, Instituto de Química y Metabolismo del Fármaco (IQUIMEFA-UBA-CONICET), Ciudad Autónoma de Buenos Aires 1113, Argentina; 3 Departamento de Química Inorgánica, Cristalografía y Mineralogía, Facultad de Ciencias, Instituto Interuniversitario en Biorrefinerías I3B, Universidad de Málaga, 29010 Málaga, Spain; ainfantes@uma.es; 4 Facultad de Matemática, Astronomía, Física y Computación, Universidad Nacional de Córdoba, Córdoba 5000, Argentina; y.garrolinck@unc.edu.ar (Y.G.-L.); belen.franzoni@unc.edu.ar (B.F.); 5 Consejo Nacional de Investigaciones Científicas y Técnicas (CONICET), Instituto de Física Enrique Gaviola (IFEG), Córdoba 5000, Argentina; 6 Consejo Nacional de Investigaciones Científicas y Técnicas (CONICET), Instituto de Investigaciones en Ciencia y Tecnología de Materiales (INTEMA), Facultad de Ingeniería, Universidad de Mar del Plata, Mar del Plata 7600, Argentina; cjperez@fi.mdp.edu.ar

**Keywords:** hydrogels, co-crosslinking, tripolyphosphate, NMR, XPS, emerging pollutants

## Abstract

In this work, the mechanical properties of hydrogels based on linear polyethyleneimine (PEI) chemically crosslinked with ethyleneglycoldiglycidyl ether (EGDE) were improved by the ionic crosslinking with sodium tripolyphosphate (TPP). To this end, the quaternization of the nitrogen atoms present in the PEI structure was conducted to render a network with a permanent positive charge to interact with the negative charges of TPP. The co-crosslinking process was studied by ^1^H high-resolution magic angle spinning (^1^H HRMAS) NMR and X-ray photoelectron spectroscopy (XPS) in combination with organic elemental analysis and inductively coupled plasma mass spectrometry (ICP-MS). In addition, the mobility and confinement of water molecules within the co-crosslinked hydrogels were studied by low-field ^1^H NMR. The addition of small amounts of TPP, 0.03 to 0.26 mmoles of TPP per gram of material, to the PEI-EGDE hydrogel resulted in an increase in the deformation resistance from 320 to 1080%, respectively. Moreover, the adsorption capacity of the hydrogels towards various emerging contaminants remained high after the TPP crosslinking, with maximum loading capacities (*q*_max_) of 77, 512, and 55 mg g^−1^ at pH = 4 for penicillin V (antibiotic), methyl orange (azo-dye) and copper(II) ions (metal ion), respectively. A significant decrease in the adsorption capacity was observed at pH = 7 or 10, with *q*_max_ of 356 or 64 and 23 or 0.8 mg g^−1^ for methyl orange and penicillin V, respectively.

## 1. Introduction

The rapid economic development has increased the demand for water, threatening global water safety. About 97.5% of the water available on the planet is salty, leaving only 2.5% fresh water to sustain human life. The low availability of water is aggravated by the uneven distribution of water that is suitable for consumption and by the increase of degraded aquifer sources. This anthropogenic deterioration has become a problem that continues to increase over time. The combination of water scarcity and poor quality of available sources is a threat to aquatic ecosystems, human health, and the economy [1]. The generation of contaminants by industries is not the problem per se, but their poor management and disposal is an issue that must be addressed. The advance of industrialization and urbanization has accelerated the discharge of contaminants into bodies of water. However, contaminants do not only remain where they are discarded but they may be spread into other surface and underground aquifers, threatening the environment, human health, and compromising water availability. According to the World Health Organization, around 2.1 billion people do not have access to safe drinking water [2]. Various strategies have been proposed for wastewater treatment, such as chemical and/or biological reduction or oxidation [3], coagulation/flocculation [4], membrane separation [5], and adsorption [6]. Among all these methods, adsorption stands out for its low cost, ease of application, and selectivity. In addition, a wide variety of porous materials can be used as adsorbents, such as clays [7], activated carbon [8], zeolites [9], and silica, among others [10].

In this context, the development of novel functional materials with good sorption properties is nowadays a major challenge to address specific problems not only in the environmental area but also in Chemistry and Biotechnology. In this sense, macromolecular chemistry is of great interest to produce processable materials with unique and valuable properties [11]. Among them, polymers offer new possibilities for scientists to create synthetic materials [12,13,14]. Particularly, our research group has studied the incorporation of coordination complexes into polymeric architectures, thus allowing the combination of the physicochemical properties of both materials [15,16,17]. Particularly, PEI-based hydrogels and chemical crosslinkers have proved to enhance the capacity of the final product to adsorb heavy metal ions [18,19,20,21,22,23] and emerging contaminants such as antibiotics and azo dyes [21,22]. However, the hydrogels’ dispersibility and their poor flocculation and separation capacity can hinder their large-scale application. Particulate fillers and chemical crosslinkers can enhance the mechanical properties to facilitate material manipulation while facilitating flocculation or filtration and allowing it to be separated from the medium [24,25,26,27]. The main methods for the formation of hydrogels are covalent and physical crosslinking, which are based on chemical reactions occurring between the functional groups of the polymer matrix and the crosslinking agent and on physical interactions such as the formation of hydrogen bonds or the generation of van der Waals or electrostatic interactions between the polymer chains and the crosslinking agent, respectively [28]. The synthesis of co-crosslinked hydrogels involves the combination of two or more types of crosslinked polymers which generate multiple crosslinking networks. These networks, resulting from the combination of different components, improve various properties of the hydrogel, such as mechanical resistance, water retention capacity, and biocompatibility, among others [29].

Few studies have assessed the modification strategies of PEI polymers in the design of materials for the adsorption of inorganic metal ions, phosphates, and dyes in wastewater. As a rule, in these studies, the branched and not the linear PEI form was employed [30,31,32,33,34]. In this regard, the embedding of the PEI polymer in the cross-linked network is the critical step to preparing a stable 3D hydrogel structure with convenient physical, mechanical, and sorption properties [21,35]. The linear PEI hydrogel synthesized by our group is a covalently EGDE-crosslinked network in which PEI contributes amino groups that can be used to generate a positive charge in the nitrogen atoms by quaternization [21,22]. By employing a negatively charged ionic crosslinking molecule, such as sodium tripolyphosphate (TPP), a co-crosslinked network can be achieved through the establishment of electrostatic interactions. In this sense, TPP is recognized as safe by the United States Food and Drug Administration (FDA) and has gained popularity for its use in various environmental and pharmaceutical applications [36]. Natural polymers such as alginate or chitosan crosslinked with TPP have been used in controlled drug delivery systems [37,38]. Moreover, different matrices have been synthesized by co-crosslinking chitosan with formaldehyde and TPP [36], as well as genipine with TPP to obtain bacterial growth inhibitors [39]. Furthermore, chitosan-poly(vinyl alcohol) blends have been used to form spherical polymeric networks using TPP as a potassium nitrate release system to be used in agricultural systems [40]. Moreover, chitosan co-crosslinked with tartrate and TPP have been used to encapsulate bovine serum albumin and tetanus toxoid [41]. Thus, the use of TPP as an ionic cross-linker can be a simple and low-cost strategy for preparing hydrogels with double crosslinking networks to improve their mechanical properties [42]. However, the increase in the cross-linking density generally results in a more rigid material with poor swelling properties [43]. For this reason, a balance is necessary not to affect both the functional groups in the hydrogel structure responsible for the uptake properties and the swelling properties, which enhance the interaction of the pollutant with the active sites of the hydrogel.

In this work, co-crosslinked hydrogels were synthesized from linear PEI-based hydrogels chemically crosslinked with EGDE using TPP as an ionic crosslinker to increase the stiffness of the hydrogel structures. The synthesis of these hydrogels was focused on environmental remediation by facilitating the removal of contaminants from water bodies. The chemical structure of the resulting hydrogels was studied at the atomic level, together with their mechanical and sorption properties. Methyl orange (MO), copper(II) ions (Cu^2+^), and the antibiotic penicillin V (PEN) in aqueous solutions were used as model contaminants. Additionally, the porosity of the PEI-EGDE and PEI-EGDE-TPP hydrogels were analyzed by low-field NMR experiments using the mobility of water molecules within the polymeric structures.

## 2. Results and Discussion

### 2.1. Synthesis and Characterization of the PEI-EGDE-TPP Hydrogels

**Scheme 1:**
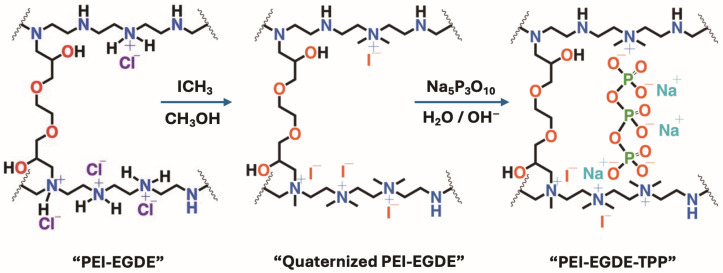
Synthetic pathways for the co-crosslinking of PEI-EGDE hydrogels (PEI-EGDE-TPP).

The hydrogel synthesis was carried out in three stages. In the first step, the PEI-EGDE hydrogel was synthesized as previously described [21,22] taking into account the nucleophilic character of the nitrogen atoms of the amino groups and the electrophilic nature of the carbon atoms of the epoxide groups in the EGDE molecules. The second stage consisted of the bimolecular nucleophilic substitution reaction between the nitrogen atoms and methyl iodide, as detailed in Scheme 1. The purpose of this stage was to alkylate the amino groups, thus ensuring that they would have positive charges independently of the pH of the medium.

Finally, the quaternized PEI-EGDE hydrogel reacted with TPP in a basic medium at a pH = 8 (Scheme 1). This procedure ensured that TPP only interacted with quaternary amino groups, preventing a reversible pH-dependent interaction with protonated amino groups. In this sense, the quaternization of the nitrogen atoms allowed the electrostatic interaction with the negative domains in the TPP molecules, thus generating a double PEI cross-linking, i.e., a network based on covalent bonds with the EGDE and a network based on ionic interactions with TPP. During the synthesis of co-crosslinked hydrogels, different proportions of TPP were used to generate three different hydrogel systems. Thus, 0.01, 0.05, and 0.1 mol of TPP were added per mol of nitrogen atom in the hydrogels. Table 1 shows the results of the organic elemental analysis of the three PEI-EGDE-TPP hydrogels, together with the phosphorus content measured through ICP-MS. While the contents of C, N, and H were not significantly affected due to their high abundance in the material, the phosphorus content increased as the concentration of TPP increased. This finding was indicative that the ionic crosslinking occurred at low amounts of TPP.

**Figure 1:**
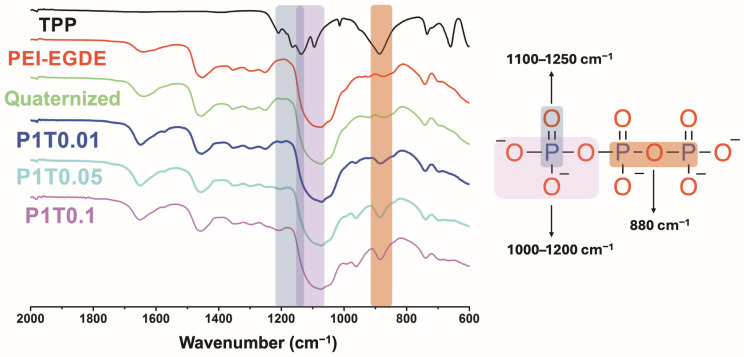
ATR-FTIR spectra for TPP, PEI-EGDE hydrogel, quaternized PEI-EGDE hydrogel, and P1T0.01, P1T0.05, and P1T0.1 co-crosslinked hydrogels.

The effect of quaternization and crosslinking with TPP on the hydrogel functional groups was first studied using ATR-FTIR spectroscopy (Figure 1). No significant differences were observed among the different treatments. All the characteristic hydrogel signals are shown, including the broad and intense absorption band around 1070 cm^−1^ attributed to stretching vibrations of C-O-C and C-N bonds [21]. For the TPP molecule, the IR absorption signals for the P=O and P-O bond stretching vibrations overlapped with the hydrogel bands at around 1100–1250 cm^−1^ and 1000–1200 cm^−1^, respectively. However, the P-O-P bending vibration at 880 cm^−1^ in PEI-EGDE-TPP hydrogels can be used to follow the co-crosslinking process as its intensity increases with the TPP content in the sample, providing evidence of the presence of TPP functional groups within the hydrogels.

**Figure 2:**
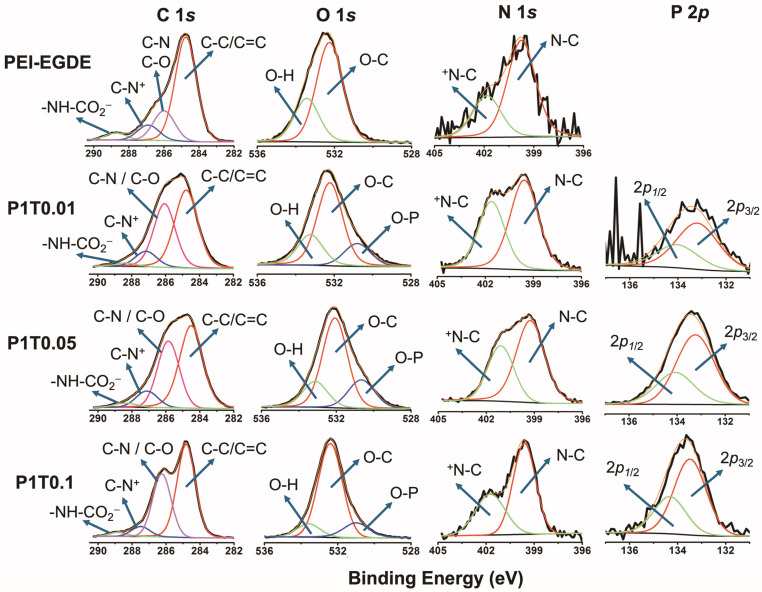
C 1*s*, O 1*s*, N 1*s,* and P 2*p* high-resolution XPS spectra for the PEI-EGE hydrogel, and P1T0.01, P1T0.05, and P1T0.1 co-crosslinked hydrogels.

To analyze the chemical composition of the material surface, XPS spectroscopy measurements were conducted. The high-resolution XPS spectra for the different elements and the results from the deconvolution analysis are presented in Figure 2 and Tables S1–S4. In the different C 1*s* core level spectra, four contributions were identified at 284.8 eV, corresponding to C-C bonds of adventitious carbon and hydrogel chains; 286.0 eV, corresponding to C-N and C-O bonds; 287.1 eV, corresponding to C-N^+^ bonds; finally, at 288.5 eV, corresponding to carbamate groups (-NH-CO_2_^−^), which can be attributed to the chemical CO_2_ adsorption on the nitrogen atoms in the hydrogel structure [44,45]. In addition, we observed that as the amount of TPP increased, an increase was also seen in the area ratio between the contributions corresponding to the C-C and C-N/C-O signals in the spectra of the co-crosslinked hydrogels (Figure 2). This phenomenon suggests that the TPP concentration significantly influences the surface chemical composition and provides valuable information on the chemical interactions and structural properties of the co-crosslinked product.

For the O 1*s* core level spectra (Figure 2 and Table S2), all the hydrogel samples shared two signals at 532.2 and 533.4 eV, corresponding to the oxygen atoms in the hydrogel structure derived from the EGDE segment (C–O) and the water molecules adsorbed on the surface of the materials, respectively [44]. Noteworthy, the signal at about 532.2 eV, associated with the C–O bond, showed an increase that was proportional to the amount of TPP used. This correlation could be due to some degree of methylation of the hydroxyl groups of the hydrogel structure. In the three co-crosslinked hydrogels, an additional signal was observed around 531.0 eV, corresponding to the O-P signals corresponding to the TPP ionically bounded to the positively charged nitrogen atoms [46]. Furthermore, the N 1*s* core level spectra (Figure 2 and Table S3) presented two signals at 399.7 eV (-N-R_2_) and 401.6 eV (-N^+^-R_3_), corresponding to the non-protonated and protonated nitrogen atoms of the PEI-EGDE hydrogel, respectively [22]. It is important to note that as the amount of TPP increased some variations were observed for both N 1*s* signals. These variations suggest that the TPP concentration significantly influences the proportion of protonated and deprotonated nitrogen atoms in the hydrogel. This phenomenon could be related to changes in the molecular structure or specific bonds induced by the presence of TPP during co-crosslinking. Particularly, the main change was detected for the alkylation process using methyl iodide in the different TPP treatments and consisted of an increment in the signal area observed at high binding energy values ascribed to the quaternary nitrogen atoms (Table S3). For all PEI-EGDE-TPP hydrogels, the N 1*s* contribution at 401.8 eV was significantly higher than that observed in the PEI-EGDE material. In addition, the P 2*p* core level spectra (Figure 2 and Table S4) showed the two contributions at 133.2 and 134.1 eV, corresponding to the binding energies for 2*p*_3/2_ and 2*p*_1/2_ of the TPP molecule [47]. It should be noted that the noise observed in the P1T0.01 sample was due to the low concentration of TPP in the sample (0.09 moles of P per gram of hydrogel, Table 1).

**Figure 3:**
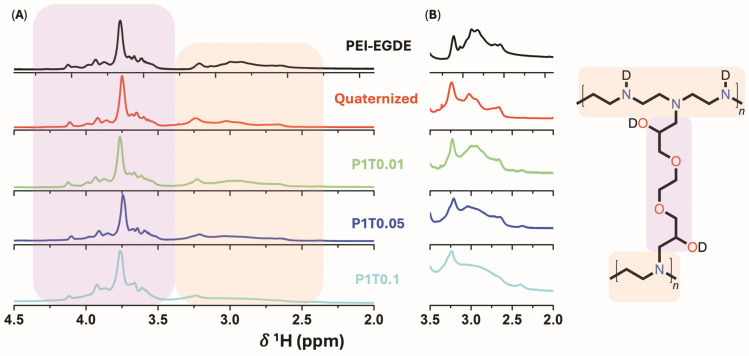
Full (**A**) and partial magnification (**B**) of the ^1^H HRMAS NMR for the PEI-EGDE, quaternized PEI-EGDE, P1T0.01, P1T0.05, and P1T0.1 hydrogels swelled in D_2_O (MAS rate = 4 kHz).

To fully characterize the material structure at the atomic level, ^1^H HRMAS NMR experiments with D_2_O were performed. Results are shown in Figure 3.

**Figure 4:**
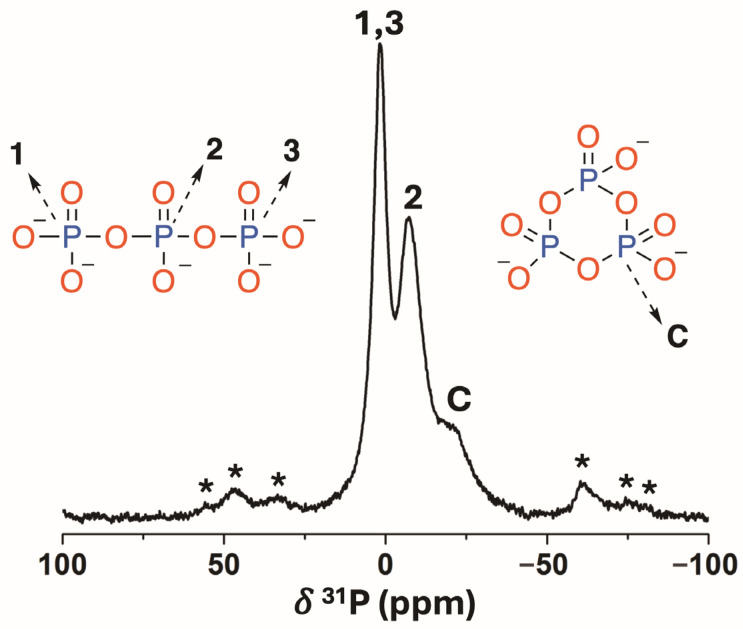
^31^P direct-polarization *ss*-NMR spectrum for P1T0.05 hydrogel (MAS rate = 15 kHz). Rotational bands are indicated with an asterisk.

In the ^1^H HRMAS NMR spectra, two distinct resonance zones were observed with proton chemical shifts (δ^1^H) around 3.5–4.3 and 2.2–3.2 ppm, corresponding to the hydrogen atoms of the polyethyleneimine and EGDE segments, respectively [21]. The main difference was observed with the expansion at a δ^1^H of 3.2–2.2 ppm (Figure 3B), in which both the alkylation of the nitrogen atoms of the PEI structure and the co-crosslinking with TPP could be indirectly inferred from the broadness of the NMR signals. This behavior may be the result of the superposition of PEI segments chemically crosslinked with EGDE and the alkylated PEI structures ionically bound to either TPP molecules or free PEI polymeric segments. Moreover, the increment in the chemical interactions between the hydrogel chains and the TPP units influenced the mobility of the polymer chains by the formation of ionic bonds, thus explaining the reduction in the swelling properties and the loss of the NMR resolution of the PEI segments around 3.5 and 2.5 ppm (Figure 3). Furthermore, a ^31^P solid-state NMR (*ss*-NMR) experiment was conducted with sample P1T0.05. The ^31^P direct polarization (DP) experiment rendered phosphorus NMR signals, evidencing the incorporation of the linear TPP into the hydrogel structure (Figure 4). In the ^31^P DP *ss*-NMR experiments, the signals at a δ^31^P of 1.7 (P_1,3_) and −7.3 ppm (P_2_) were assigned to the phosphorus atoms of the linear TPP structure (Figure 4) [48]. On the other hand, the signal at a δ^31^P of −20.9 ppm was associated to some degree with the cyclic phosphate structures originating from linear TPP during the thermal drying of the hydrogel material carried out for *ss*-NMR studies [48]. Particularly, ^31^P cross-polarization experiments (CP-MAS) did not provide signals at the same number of scans (NS = 2000). In the ^31^P CP-MAS experiment, the low amount of phosphorus and the uneven solid structure in the P1T0.05 sample did not allow the correct cross-polarization process from ^1^H to ^31^P in the co-crosslinked hydrogel.

**Figure 5:**
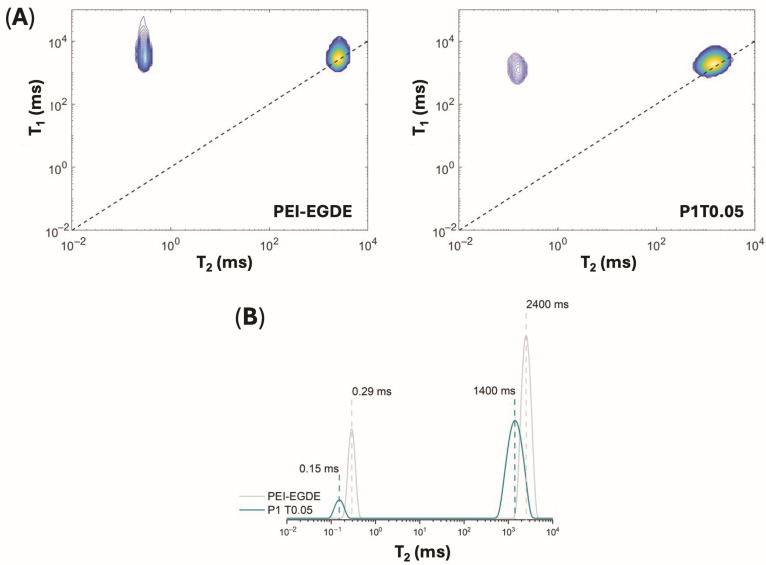
T_1_–T_2_ maps (**A**) and T_2_ projections for the indicated hydrogels (**B**).

Additionally, low-field NMR experiments were conducted with PEI-EGDE and P1T0.05 hydrogels. The T_1_-T_2_ maps are shown in Figure 5A. Two different populations are easily distinguished in each map, which are characterized by different T_2_ relaxation rates. A shorter T_2_ component, with relaxation times in the hundreds of microseconds, likely represents structural water molecules bound to the polymer walls. The population with longer T_2_ relaxation times appeared above the T_1_ = T_2_ diagonal in both samples. This phenomenon is expected in two cases: (a) when free bulk fluids or fluids confined in large cavities (compared to diffusion mobility) are present, and (b) when fluids are confined in such a way that the molecular mobility is physically restricted and consequently both T_1_ and T_2_ decrease equally from their free-fluid values [49,50,51]. For the PEI-EGDE hydrogel, T_1_ = T_2_ = 2.4 s. This value is in agreement with those expected for free water molecules. This indicates that the mobility of water molecules in this sample is not restricted mobility. However, as the hydrogel structure was modified, the T_1_/T_2_ value decreased, accounting for a more structured matrix where water diffusion is restricted. These variations are more clearly identified in Figure 5B, where the T_2_ projection for the P1T0.05 sample is plotted compared to the projection for the PEI-EGDE hydrogel. After the chemical modification with TPP, the T_2_ population that appeared at longer T_2_ values shifted towards lower T_2_ values, reflecting the confinement of water molecules induced by the increase in the mechanical resistance of the PEI-EGDE hydrogels after the co-crosslinking with TPP (P1T0.0.5).

### 2.2. Swelling and Mechanical Properties

**Figure 6:**
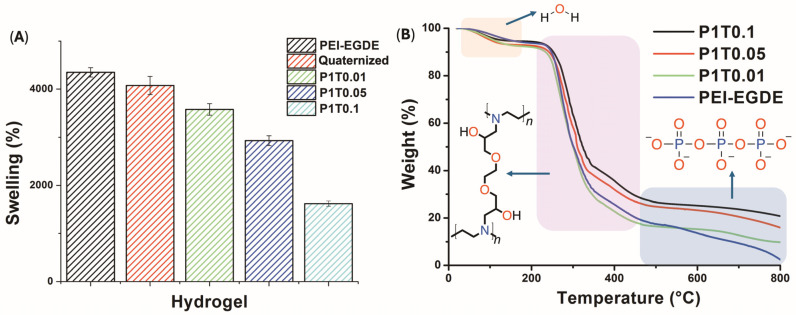
Swelling capacities (**A**) and TGA curves (**B**) for the PEI-EGDE, quaternized PEI-EGDE, P1T0.01, P1T0.05, and P1T0.1 hydrogels.

Swelling is one of the fundamental properties of hydrogels, as it indicates their capacity to retain water molecules or other solvents within their network. Upon quaternization of the nitrogen atoms, a significant decrease in the swelling was observed (Figure 6A). It was observed that as the amount of TPP increased, swelling capacities were strongly reduced. This can be attributed to the fact that, as the crosslinking density increases, the polymer chains become more restricted in terms of mobility and capacity to retain water molecules in their structure. In this sense, another important property of hydrogels, especially when applied as contaminant adsorbents, is the pH of the point of zero charge (pH_pzc_). The pH_pzc_ is the pH at which the charge of the hydrogel is electrically neutral. This value is crucial in determining how the hydrogel will interact with its environment, as the charged molecules will be either attracted or repelled by the surface of the hydrogel depending on its charge and the pH of the medium. Figure S1 presents the pH_pzc_ values obtained as a function of the crosslinking degree with TPP. Notably, the addition of TPP generated a decrease in the pH_pzc_ value. This can be attributed to two factors, firstly, the quaternization of amino groups may change the nature of the functional groups in the hydrogel, affecting the electrostatic interactions and thus the pH_pzc_ value. Secondly, TPP is a polyanion with a net negative charge. By adding TPP to the hydrogel, a higher negative charge density is introduced in the polymer network. This increases the number of ionizable groups (in this case, protonated amino groups) needed to neutralize the negative charge of TPP. As a result, a lower pH is required to achieve total neutralization, which results in a lower pH_pzc_ value.

To study the effect of the hydrogel crosslinking on their thermal properties, a thermogravimetric analysis (TGA) was conducted, and the results are shown in Figure 6B. The mass loss of the hydrogel samples took place in two steps. The first step spanned from 10 to 200 °C, with a loss of about 5% of the initial mass, and this loss was attributed to the loss of water molecules adsorbed in the pores of the hydrogels. The second step spanned from 250 to 480 °C, with a loss of 69, 64, 76, and 79% of the initial mass for P1T0.1, P1T0.05, P1T0.01, and PEI-EGDE, respectively. This loss was attributed to the oxidation of the hydrocarbon structure of the hydrogels [22]. The results of the TGA studies showed that the hydrogel with higher TPP content presented a higher degradation temperature, which was attributed to the higher crosslinking density that occurred when TPP was incorporated into the polymer network. The increase in the crosslinking density limited the mobility of the polymer chains, making it difficult to break chemical bonds during thermal degradation [41,52]. The degradation temperature decreased along with the decrease in the amount of TPP, being quite similar for the P1T0.01 and PEI-EGDE hydrogels. In the case of co-crosslinked hydrogels and for temperatures above 500 °C, the residue was associated with the molecules of TPP, which is a substance with high thermal stability [53]. In contrast, for the TPP-free hydrogel, no residue was obtained at the end of the TGA analysis (Figure 6B).

**Figure 7:**
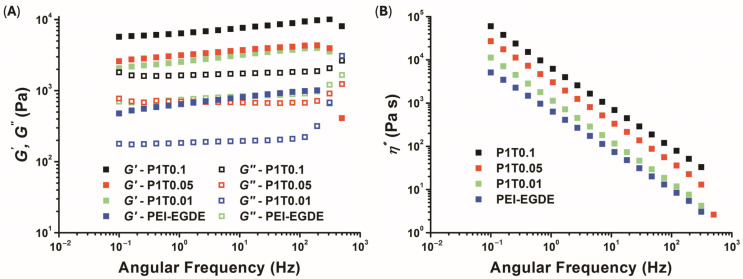
Rheological properties of PEI-EGDE, P1T0.01, P1T0.05, and P1T0.1 hydrogels: storage and loss moduli (**A**) and complex viscosity (**B**).

To complete the analysis of the properties of the co-crosslinked hydrogels, the change in the response to the deformation of materials caused by the co-crosslinking procedure was studied. To this end, several rheological parameters were measured, such as the storage (*G*′) and loss (*G*″) moduli and the complex viscosity. Figure 7A shows *G*′ and *G*″ values for the different polymeric materials. All the hydrogels, within the frequency range studied (0.1–500 Hz), under a deformation of 1%, presented a linear viscoelastic response. Furthermore, for all the analyzed hydrogels, *G*′, which is related to the elastic component, was greater than *G*″, which is related to the viscous component. This behavior indicated that the hydrogels behaved in a similar way to elastic solids, implying that hydrogels maintained their capacity to recover their original form after deformation [54].

On the other hand, the values of *G*′ and *G*″ increased together with the content of TPP, which indicates that TPP acted as a physical crosslinking agent by reinforcing the density of the crosslinked hydrogels and their resistance to deformation [55]. The capacity of hydrogels to resist deformation and maintain their structure against external forces is essential for their effectiveness in the removal of contaminants from water bodies since they can maintain their structural integrity even in flowing or agitated waters. The addition of TPP to the hydrogel resulted in an increase in the deformation resistance of 320, 430, and 1080% for P1T0.01, P1T0.05, and P1T0.1 samples, respectively.

The complex viscosity (η*), analyzed as a function of the angular frequency for the hydrogels, is related to the stability of the hydrogel structure [56]. For all materials, η* decreased linearly as the frequency increased, which is indicative that the materials behaved as non-Newtonian fluids with a pseudo-plastic behavior (Figure 7B) [57]. The η* values increased along with the TPP content. This finding is further evidence that TPP, by acting as a physical crosslinker, increased the rigidity of the hydrogel networks, allowing a greater capacity of materials to withstand stress. As observed in Section 2.1 (Synthesis and Characterization of the PEI-EGDE-TPP Hydrogels), the amount of TPP in the three materials was low, as inferred from the elemental analysis (2.8–24.2 mg of phosphorus per gram of material, Table 1). However, as studied in this section, the properties of the hydrogels were significantly enhanced by the addition of TPP as an ionic crosslinker. As the content of the TPP increased, the materials presented a more rigid structure with a lower capacity to retain water molecules and greater resistance to deformation in comparison with the PEI-EGDE hydrogel. Similar improvements in the mechanical properties of the PEI-EGDE hydrogel were attained by using higher amounts of the EGDE molecules (chemical crosslinker) [21] or by using the MOF DUT-67 as a particular filler during the synthesis of the materials [22]; however, a significant reduction in the swelling capacities and an increase in the synthetic procedure cost were observed. In this sense, the mechanical properties were improved using ionic crosslinking with TPP without affecting crucial properties such as swelling and sorption capacities, which will be discussed in the following section.

### 2.3. Sorption Studies

After studying the properties of the co-crosslinked hydrogels, P1T0.01 was selected to perform adsorption studies since with a lower amount of TPP, an improvement in the mechanical properties was achieved due to the increase in *G*′ of approximately 58% (Figure 7). The swelling, which is a property with a significant impact on the adsorption capacity of a material, did not decrease as much when compared to P1T0.05 and P1T0.1. Additionally, it was the material in which the lowest amount of TPP was used, thus reducing the production cost. Hydrogels can adsorb contaminants present in different media, such as water. These contaminants can be organic compounds, heavy metal ions, azo-dyes, and antibiotics, among others.

**Figure 8:**
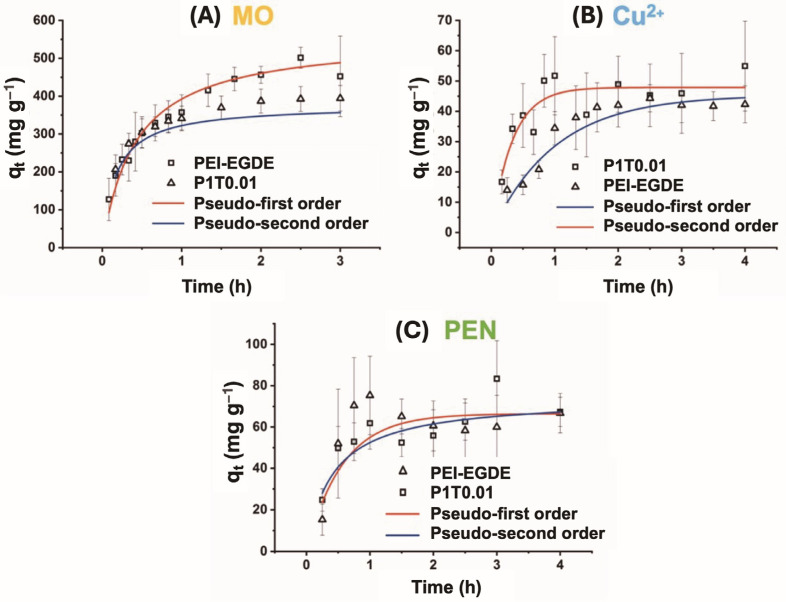
Adsorption kinetics of MO (**A**), Cu^2+^ ions (**B**) and PEN (**C**) by the PEI-EGDE and P1T0.01 hydrogels together with the kinetic model fittings.

To investigate the adsorption rate of MO, Cu^2+^, and PEN by P1T0.01 and PEI-EGDE hydrogels, kinetic studies were conducted in the range of 3 and 4.5 h. Figure 8 shows the data corresponding to the adsorption of MO at pH = 6, Cu^2+^ at pH = 4, and PEN at pH = 4. In addition, Table 2, Table 3 and Table 4 show the kinetic model values for adsorption of the different contaminants used in the experiments. In general, the adsorption process for both hydrogels with the three contaminants exhibited a fast kinetics, reaching equilibrium after 60–120 min. This was probably due to the open porous structure of the swollen hydrogel.

Regarding MO, the best fit for the experimental data of both materials was the pseudo-second-order model. This means that the adsorption rate was controlled by the chemical interaction between the hydrogel and the contaminant [58]. MO is an anionic contaminant, and the hydrogel has a positive charge density and during the adsorption process, electrostatic interactions between both species must play a key role. On the other hand, the addition of the TPP generated a decrease in the adsorption capacity of the hydrogel (Table 2). This decrease can be attributed to two factors: one, was related to the lower swelling capacity and a less accessible structure in P1T0.01 in comparison with the PEI-EGDE hydrogel; the second factor is that TPP has a net negative charge, which generates electrostatic repulsion with MO molecules, which also have a negative charge at pH = 4.0. This additional electrostatic repulsion further hinders the adsorption of MO on the crosslinked materials. In this sense, MO molecules were restricted to reach the positively charged nitrogen atoms in the co-crosslinked hydrogels where the adsorption process occurs, thus reducing the sorption capacities of the P1T0.01 hydrogel.

In the case of Cu^2+^ ions, their interaction with the nitrogen atoms of the secondary amine groups was favored, as stable coordinated complexes are formed [12,21,23]. For the P1T0.01 hydrogel, the experimental data are appropriately described by the pseudo-second-order kinetic model, and for the PEI-EGDE hydrogel, by the pseudo-first-order model. However, for both hydrogels, the values of χ^2^ and RSS (Table 3) for both models were similar. Therefore, the adsorption rate was controlled by chemical and physical interactions between the hydrogel and the contaminant. The addition of TPP increased the adsorption capacity of the hydrogel, which can be attributed to the fact that phosphate groups can also coordinate Cu^2+^ ions [59].

As for the adsorption of PEN, at pH = 4.0, the amino groups in the hydrogel structure are protonated (positively charged in sites that have not been fully quaternized), while most carboxylic acid groups of PEN are deprotonated (negatively charged), which favors the electrostatic interaction between the functional groups of the material and the contaminant. As with the Cu^2+^ ions, the experimental data are adequately described by pseudo-first and pseudo-second-order models, indicating that the adsorption process was governed by both chemical and physical interactions between the hydrogel and the contaminant. Regarding the adsorption capacities, no significant changes were observed after the addition of TPP to the hydrogel (Table 4).

**Figure 9:**
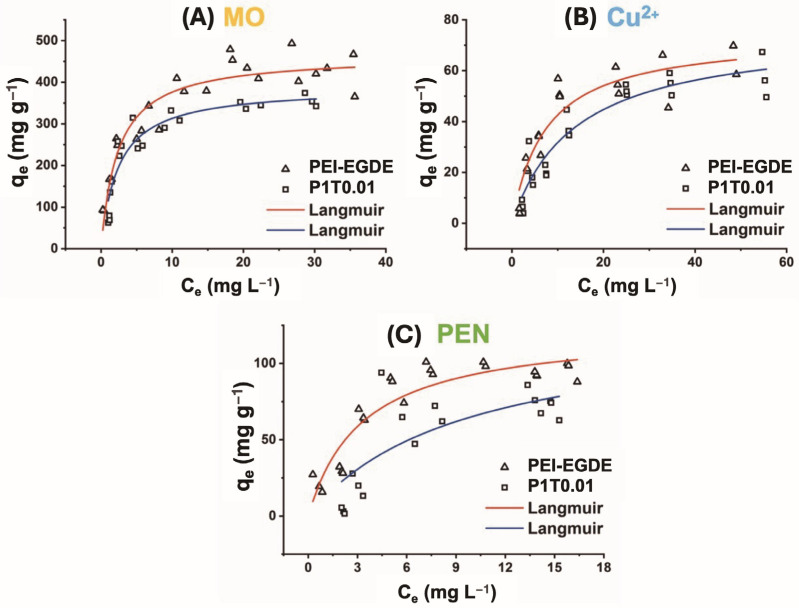
Adsorption isotherms of MO (**A**), Cu^2+^ ions (**B**) and PEN (**C**) by the PEI-EGDE and P1T0.01 hydrogels together with the fittings to the Langmuir model.

Moreover, adsorption isotherm experiments were conducted to provide information on the interaction between the adsorbent and the contaminant under equilibrium conditions, allowing us to characterize the interaction affinity. In addition, by adjusting the experimental data obtained from the adsorption experiments to mathematical models, the mechanism and maximum adsorption capacity could be determined. Experimental data related to adsorption isotherms and fitting parameters are shown in Figure 9 and Table 5, respectively. For the three contaminants (MO, Cu^2+^, and PEN), the experimental isotherm data for the PEI-EGDE and P1T0.01 hydrogels properly fitted the Langmuir model. This indicates that MO, PEN, and Cu^2+^ ions may form a monolayer on the surface of the hydrogels, favoring the chemical adsorption process.

The experimental values of *q*_max_ for MO were 356.2 and 430.6 mg g^−1^ for P1T0.01 and PEI-EGDE hydrogels, respectively, while the calculated values were 388.2 and 464.3 mg g^−1^. For Cu^2+^ ions, the experimental values of *q*_max_ were 55.1 and 60.6 mg g^−1^, with calculated values of 74.6 and 76.6 mg g^−1^ for P1T0.01 and PEI-EGDE hydrogels, respectively. Finally, the experimental values of *q*_max_ for PEN were 77.7 and 95.7 mg g^−1^, with calculated values of 127.4 and 123.9 mg g^−1^ for P1T0.01 and PEI-EGDE hydrogels, respectively. All experimental and calculated values showed differences that were within the range of 4–11%, which is considered acceptable. Aside from the lower *q*_max_ of MO adsorption of P1T0.01 in comparison with the PEI-EGDE hydrogels, *q*_max_ values for Cu^2+^ and PEN adsorption were found to be similar for both hydrogels. This would imply that the co-crosslinking treatment is not hindering the hydrogel adsorption capacity. Nevertheless, in the case of MO, as stated above, the generated electrostatic repulsion may lower the adsorption capacity of the material (Table 5).

Furthermore, the effect of pH on the uptake properties of the contaminants was studied, since this is a factor that plays a crucial role in the adsorption process by influencing the behavior of both the adsorbent and the adsorbate. To investigate the impact of pH on the adsorption capacities of PEI-EGDE and P1T0.01 materials, adsorption experiments were carried out with MO and PEN at pH 4, 7 and 10. The results of these experiments are shown in Table 6. In the case of Cu^2+^ ions, the effect of pH on the adsorption process could not be studied, as copper precipitates as copper hydroxide at pH ≥ 5.2 [60].

It is worth noting that for both materials, as pH values increased, a significant decrease in the adsorption capacity was observed. This result was expected due to the nature of the hydrogels and contaminants involved. At pH = 4, most of the MO (*pKa* = 4.3) and PEN (*pKa* = 2.7) molecules are negatively charged and attracted by the protonated amino groups of the nitrogen atoms that have not been fully quaternized in the polymer network, allowing the material to interact effectively with MO and PEN molecules, which are negatively charged. As the pH increased, the amino groups of the polymer became deprotonated, leading to a reduction of the capacity of the hydrogels to electrostatically interact with the molecules of interest.

## 3. Conclusions

Polyethyleneimine hydrogels with a double crosslinking network, one with the covalent crosslinking agent EGDE and the other with the ionic agent TPP, were synthesized. In terms of molecular structure, the presence of TPP functional groups within co-crosslinked hydrogels was confirmed by spectroscopic techniques (HRMAS NMR, IR, and XPS) and elemental analysis. The incorporation of TPP increased the cross-linking density, resulting in a decrease in swelling and an increase in the degradation temperature and deformation resistance of the materials. The improvement of the mechanical properties achieved with small amounts of TPP rendered co-crosslinked hydrogels that were easier to manipulate, facilitating flocculation and filtration procedures during the adsorption processes in comparison with PEI-EGDE hydrogel. In this sense, the deformation resistance increased from 320 to 1080% with 0.03 to 0.26 mmol of TPP per gram of material, respectively. The use of TPP-crosslinked hydrogels was less expensive than the use of MOF particles as fillers and with similar results. Moreover, low-field NMR studies revealed that TPP-crosslinked hydrogels had a more structured matrix and presented a concomitant reduction of the mobility of water molecules.

Regarding adsorption studies, it was observed that the adsorption capacity of contaminants such as MO, Cu^2+^, and PEN was slightly affected by the presence of TPP. While the adsorption of MO and PEN decreased due to the generation of additional electrostatic repulsions, the adsorption capacity of Cu^2+^ ions was found to be increased, which can be attributed to the establishment of specific chemical interactions with the TPP phosphate groups. The adsorption of MO, Cu^2+^, and PEN by hydrogels involved both physical and chemical adsorption processes. The Langmuir model provided the best fit for the adsorption isotherms, suggesting monolayer adsorption of contaminants on the hydrogel surface. The adsorption capacity decreased with the increasing pH for MO and PEN.

## 4. Materials and Methods

### 4.1. Materials

The linear polyethyleneimine hydrochloride polymer with a nitrogen content of 14.8% (87 kDa as free base) was synthesized as described previously [12]. Details related to this section can be found in the Supplementary Material (Experimental S1).

### 4.2. Synthesis of Co-Crosslinked Hydrogels

In an Erlenmeyer flask, 320 mg of PEI.HCl (~1.7 mmol of nitrogen) was dissolved in 6.4 mL of deionized water and 0.80 mL (2.6 mmol) of EGDE. The solution was heated by stirring at 90 °C for 140 min. After heating, the formed material was swelled in methanol and granulated using a 1000-µm sieve to homogenize the particle size and facilitate washing. The material was washed three times with 50 mL of methanol, followed by drying at room temperature. Then, 500 mg of dry hydrogel, 250 mL of methanol, and 0.250 mL of methyl iodide (CH_3_I) (4.02 mmol) were added to a beaker to methylate the hydrogel. The reaction was kept under agitation for 24 h. After that, the quaternized hydrogel was washed three times with 50 mL methanol and dried at room temperature. After that, 350 mg of dry quaternized hydrogel (P1 sample) was mixed with 250 mL of deionized water using 0.1 M KOH to adjust to pH = 8. Then, the necessary amount of TPP was added to form three samples with different levels of co-crosslinking: 5.3 mg (14.4 mol), 26.7 mg (72.6 mol), and 53.3 mg (145 mol). These samples were named P1T0.01, P1T0.05, and P1T0.1, respectively. Subsequently, an acid treatment was carried out with 0.5 mL of a 6 M HCl solution for 1 h at room temperature to hydrolyze the remaining epoxides from the EGDE. Finally, the materials were washed three times with a 10 mM dibasic potassium phosphate buffer solution to achieve a pH~6. A final wash was performed with deionized water.

### 4.3. Characterization Techniques

Details related to these techniques can be found in the Supplementary Material (Experimental S2).

### 4.4. Kinetic and Adsorption Capacity Assessment

Details related to these experiments can be found in the Supplementary Material (Experimental S3 and S4).

## Figures and Tables

**Table 1:** Organic elemental analysis and phosphorus content for the P1T0.01, P1T0.05, and P1T0.1 hydrogels.

Sample	%C	%H	%N	mmol P g^−1^	mmol TPP g^−1^
P1T0.01	40.5	7.2	6.8	0.09	0.03
P1T0.05	39.5	7.3	6.8	0.30	0.10
P1T0.1	38.7	7.2	7.0	0.78	0.26

**Table 2:** Kinetic model parameters for MO adsorption.

		PEI-EGDE		P1T0.01	
Model	Parameter	Value	Goodness-of-Fit	Value	Goodness-of-Fit
Pseudo 1st order	*q_e_* (mg L^−1^)	468 ± 14	χ^2^ = 0.6	334 ± 9	χ^2^ = 0.7
	*k*_1_ (h^−1^)	2.0 ± 0.2	RSS = 7.4	4.59 ± 0.47	RSS = 5.2
Pseudo 2nd order	*q_e_* (mg L^−1^)	557 ± 16	χ^2^ = 0.3	377 ± 4	χ^2^ = 0.05
	*k*_2_ (g mg^−1^ h^−1^)	(42.8 ± 6.1) × 10^−4^	RSS = 3.2	(156 ± 9) × 10^−4^	RSS = 0.5
Elovich	*α* (mg g^−1^ h^−1^)	0.6 ± 0.1	χ^2^ = 1.8	1.27 ± 0.27	χ^2^ = 1.4
	*β* (g mg^−1^)	(27.9 ± 0.9) × 10^−4^	RSS = 10.9	(41.2 ± 3.2) × 10^−4^	RSS = 11.1
Modified Freundlich	*k_F_* (L g^−1^ h^−1^)	6.1 ± 0.1	χ ^2^ = 0.3	9.5 ± 0.2	χ^2^ = 1.4
	*m*	3.94 ± 0.12	RSS = 2.1	4.5 ± 0.4	RSS = 7.3

**Table 3:** Kinetic model parameters for Cu^2+^ adsorption.

		PEI-EGDE		P1T0.01	
Model	Parameter	Value	Goodness-of-Fit	Value	Goodness-of-Fit
Pseudo 1st order	*q_e_* (mg L^−1^)	45.4 ± 2.6	χ^2^ = 0.6	47.9 ± 3.0	χ^2^ = 0.4
	*k*_1_ (h^−1^)	0.99 ± 0.14	RSS = 5.0	3.01 ± 0.5	RSS = 3.7
Pseudo 2nd order	*q_e_* (mg L^−1^)	58.9 ± 5.7	χ^2^ = 0.7	56.1 ± 5.0	χ^2^ = 0.5
	*k*_2_ (g mg^−1^ h^−1^)	(15.2 ± 5.0) × 10^−3^	RSS = 6.2	(60.1 ± 20.4) × 10^−3^	RSS = 4.5
Elovich	*α* (mg g^−1^ h^−1^)	0.34 ± 0.17	χ^2^ = 1.1	0.8 ± 0.3	χ^2^ = 1.4
	*β* (g mg^−1^)	0.034 ± 0.002	RSS = 9.6	0.03 ± 0.004	RSS = 12.4
Modified Freundlich	*k_F_* (L g^−1^ h^−1^)	1.9 ± 0.1	χ^2^ = 1.0	1.3 ± 0.09	χ^2^ = 1.0
	*m*	2.2 ± 0.3	RSS = 9.4	3.4 ± 0.7	RSS = 8.7

**Table 4:** Kinetic model parameters for PEN adsorption.

		PEI-EGDE		P1T0.01	
Model	Parameter	Value	Goodness-of-Fit	Value	Goodness-of-Fit
Pseudo 1st order	*q_e_* (mg L^−1^)	63 ± 5	χ^2^ = 0.6	63 ± 3	χ^2^ = 0.6
	*k*_1_ (h^−1^)	1.8 ± 0.5	RSS = 4.2	2.2 ± 0.4	RSS = 4.2
Pseudo 2nd order	*q_e_* (mg L^−1^)	80 ± 11	χ^2^ = 0.8	74 ± 5	χ^2^ = 0.5
	*k*_2_ (g mg^−1^ h^−1^)	(21.9 ± 13.1) × 10^−3^	RSS = 5.9	0.03 ± 0.01	RSS = 3.9
Elovich	*α* (mg g^−1^ h^−1^)	0.5 ± 0.5	χ^2^ = 1.8	0.9 ± 0.4	χ^2^ = 1.3
	*β* (g mg^−1^)	0.02 ± 0.003	RSS = 12.6	0.021 ± 0.003	RSS = 8.9
Modified Freundlich	*k_F_* (L g^−1^ h^−1^)	6.1 ± 0.7	χ^2^ = 1.5	6.3 ± 0.4	χ^2^ = 0.9
	*m*	2.88 ± 1.0	RSS = 10.5	3.4 ± 0.7	RSS = 6.4

**Table 5:** Model fitting parameters for MO, Cu^2+^, and PEN adsorption isotherms.

MO					
		PEI-EGDE		P1T0.01	
Model	Parameter	Value	Goodness-of-Fit	Value	Goodness-of-Fit
Langmuir	*q_m_* (mg g^−1^)	464 ± 18	χ^2^ = 1660	388 ± 17	χ^2^ = 1134
	*k_a_* (L mg^−1^)	0.4 ± 0.08	RSS = 34,873	0.5 ± 0.07	RSS = 21,542
Freundlich	*k*	185 ± 16	χ^2^ = 2142	146 ± 15	χ^2^ = 2310
	*n*	0.2 ± 0.03	RSS = 45,000	0.3 ± 0.04	RSS = 43,895
**Cu^2+^**					
		**PEI-EGDE**		**P1T0.01**	
**Model**	**Parameter**	**Value**	**Goodness-of-Fit**	**Value**	**Goodness-of-Fit**
Langmuir	*q_m_* (mg g^−1^)	74 ± 6	χ^2^ = 60.4	76 ± 6	χ^2^ = 44.5
	*k_a_* (L mg^−1^)	0.1 ± 0.03	RSS = 1027	0.07 ± 0.02	RSS = 846
Freundlich	*k*	15.2 ± 3.0	χ^2^ = 108	10.5 ± 2.05	χ^2^ = 67.1
	*n*	0.4 ± 0.06	RSS = 1841	0.4 ± 0.06	RSS = 1276
**PEN**					
		**PEI-EGDE**		**P1T0.01**	
**Model**	**Parameter**	**Value**	**Goodness-of-Fit**	**Value**	**Goodness-of-Fit**
Langmuir	*q_m_* (mg g^−1^)	123 ± 9	χ^2^ = 129	127 ± 42	χ^2^ = 373
	*k_a_* (L mg^−1^)	0.30 ± 0.07	RSS = 2712	0.1 ± 0.07	RSS = 5591
Freundlich	*k*	37 ± 5	χ^2^ = 209	15.4 ± 6.2	χ^2^ = 421
	*n*	0.38 ± 0.05	RSS = 4387	0.6 ± 0.2	RSS = 6322

**Table 6:** Influence of pH on MO and PEN adsorption capacities of PEI-EGDE and P1T0.01 hydrogels.

	pH = 4		pH = 7		pH = 10	
Hydrogel	MO (mg g^−1^)	PEN (mg g^−1^)	MO (mg g^−1^)	PEN (mg g^−1^)	MO (mg g^−1^)	PEN (mg g^−1^)
PEI-EGDE	648 ± 27	94 ± 7	430 ± 64	25 ± 3	78 ± 5	1.6 ± 1.0
P1T0.01	512 ± 32	77 ± 7	356 ± 17	23 ± 4	64 ± 8	0.8 ± 0.5

## Data Availability

The original contributions presented in this study are included in the article/Supplementary Materials. Further inquiries can be directed to the corresponding authors.

## Supplementary Materials

The following supporting information can be downloaded at: https://www.mdpi.com/article/10.3390/gels10120790/s1, materials (Experimental S1), characterization techniques (Experimental S2); kinetic and adsorption capacity assessment (Experimental S3) and kinetic and isotherm models (Experimental S4); Tables S1–S4. Fitting parameters from XPS data; Figure S1. *pH_pzc_* for the PEI-EGDE, P1T0.01, P1T0.05 and P1T0.1 hydrogels. References [61,62,63,64,65,66,67,68,69,70,71,72,73,74,75,76].
