# Supplementary material for: Ionic Crosslinking of Linear Polyethyleneimine Hydrogels with Tripolyphosphate
Source: Gels. 2024 Dec 3;10(12):790. doi: 10.3390/gels10120790 (PMC11675353; doi:10.3390/gels10120790)
Supplement: Supplementary file 1 [file gels-10-00790-s001.zip › gels-3312444-supplementary.pdf]

gels MDPI

'Supplementary Material'

Article

Ionic Crosslinking of Linear Polyethyleneimine Hydrogels with Tripolyphosphate

Luis M. Araque [1,2], Antonia Infantes-Molina [3], Enrique Rodríguez-Castellón [3,*], Yamila Garro-Linck [4,5], Belén Franzoni [4,5], Claudio J. Pérez [6], Guillermo J. Copello [1,2] and Juan M. Lázaro-Martínez [1,2,*]

Table of Contents:

Content	*Page*
Experimental S1. Materials	1
Experimental S2. Characterization techniques	1
Experimental S3. Kinetic and adsorption capacity assessment	2
Experimental S4. Kinetic and Isotherm models	3
Table S1. Fitting parameters from C 1s XPS data	4
Table S2. Fitting parameters from O 1s XPS data	4
Table S3. Fitting parameters from N 1s XPS data	5
Table S4. Fitting parameters from P 2p XPS data	5
Figure S1. pH_{pzc} for the hydrogels.	6
References	7

Experimental S1. ***Materials***

Analytical grade EGDE (TCI—Portland, Oregon, USA) and sodium tripolyphosphate (TPP), hydrochloric acid, sodium or potassium hydroxide, methyl iodide, methyl orange (MO), penicillin V (PEN), phosphate buffer, sodium chloride, copper(II) sulphate, and deuterium oxide (D_2O) (Merck—Rahway, New Jersey, USA) were of analytic grade and used without further purification.

Experimental S2. Characterization techniques

General. ATR-FTIR, and FT-Raman spectra were recorded on a Nicolet iS50 spectrometer (Thermo Scientific, Waltham, Massachusetts, USA) using a one-reflection diamond crystal. High-resolution magic angle spinning (HRMAS) NMR and ^{31}P solid-state NMR spectra were acquired with a Bruker Avance-III HD spectrometer (Billerica, Massachusetts, USA) equipped with a 14.1 T narrow bore magnet. The thermogravimetric analysis (TGA) was recorded in a TGA-50 Shimadzu (Kawasaki, Japan). X-ray photoelectron spectroscopy (XPS) analysis was carried out with a Physical Electronics Versa-Pro II (Chanhassen, Minnesota, USA) operating with a monochromatic X-ray source Al (k-alpha) of photons at 1486 eV under ultra-high vacuum using a pressure of 10^{-6} Pa. The viscoelastic behavior was determined with an Anton Paar rotational rheometer MCR-301 (Graz, Austria). Organic elemental analysis was performed in a CE440 Elemental Analyser device (Coventry, United Kingdom). Phosphorus determinations were carried out with an Inductively Coupled Plasma Atomic Emission Spectrometer (ICP-OES), Shimadzu 9000 ((Kawasaki, Japan).

ATR-FTIR spectra were recorded on a Nicolet iS50 spectrometer using a one-reflection diamond crystal with 64 scans at a resolution of 4 cm^{-1}. All samples were previously oven-dried at 60°C for 24 h.

HRMAS NMR and *ss*-NMR spectra were acquired with a Bruker Avance-III HD spectrometer equipped with a 14.1 T narrow bore magnet operating at Larmor frequencies of 600.09 and 242.92 MHz for ^1H and ^{31}P, respectively. Crosslinked and co-crosslinked

hydrogels were studied by ^{1}H NMR HRMAS by packing the D_2O-swelled sample into a 4 mm ZrO_2 HRMAS rotor with a 50 µL spherical insert. The sample was spun at a MAS rate of 4 kHz. A pre-saturation pulse (zgpr) was used for water-suppression in the ^{1}H experiments. Powdered sample was packed into 3.2 mm ZrO_2 rotors and rotated at room temperature at MAS rates of 15 kHz. ^{31}P *ss*-NMR spectrum was recorded using high powered decoupling (hpdec) experiment at a MAS rate of 15 kHz in 3.2-mm MAS probe. Chemical shifts for ^{31}P and ^{1}H (expressed in ppm) are relative to H_3PO_4 85% and $(CH_3)_4Si$, respectively.

The TGA was recorded in a TGA-50 Shimadzu. Samples were previously oven-dried at 60°C for 24 h. The analysis was performed from 25 to 800 °C in a nitrogen atmosphere with a heating rate of 10°C min^{-1}.

XPS analysis was used to obtain quantitative and chemical state information on the surface of the materials, and was carried out with a Physical Electronics Versa-Pro II (Chanhassen, Minnesota, USA) operating with a monochromatic X-ray source Al (k-alpha) of photons at 1486 eV under ultra-high vacuum using a pressure of 10^{-6} Pa. The XPS experimental results were analyzed using a 0.651 eV Au $4f_{7/2}$ line of full width at half maximum.

The swelling behavior of composite hydrogels was studied by the gravimetric method carried out in distilled water at 25°C in triplicate. In each experiment, the oven-dried hydrogel was immersed in water for 24 h. The swelled sample was then separated from the solution and weighed after previous surface water removal with filter paper. Finally, the swelled samples were oven-dried at 60°C until constant weight was obtained. The swelling capacity (S) was calculated using the following equation:

$$S(\%) = \frac{M_s - M_d}{M_d} \times 100 \qquad (1)[61]$$

where M_s and M_d are the swelled and dried sample weight, respectively.

The pH at the point of zero charge (pH_{pzc}) of the hydrogels was determined by the pH drift method.[62] The pH of NaCl solutions (10 mM) was adjusted over a range of 4 – 8 by adding either 0.1 M HCl or 0.1 M NaOH. Then, 50 mg of the swelled samples were immersed into 10 mL of each solution, incubated for 24 h at room temperature, and the final pH was measured. The pH value at which the curve of the final pH crosses the pHinitial=pHfinal lines is the pH_{pzc}.

The viscoelastic behavior was determined with an Anton Paar rotational rheometer MCR-301 (Graz, Austria). Parallel plates (d=25 mm) were used for the frequency sweep test from 0.1 to 500 s^{-1} using a strain value of 1% at 25°C. Measures fell within the lineal viscoelastic range, as assessed previously.

Low field NMR experiments were carried out in a Bruker minispec mq20 (Billerica, Massachusetts, USA), with an operational frequency of 20 MHz for ^{1}H nuclei. The determination of NMR longitudinal (T_1) and transverse (T_2) relaxation times is traditionally used to probe confined fluid properties. Brownstein and Tarr [63] have demonstrated that, for confined fluids, both relaxation methods are proportional to the volume-to-surface ratio of the confining pores. On the other hand, surface interactions generally affect the transverse relaxation times more strongly [64]. Consequently, bidimensional T_1-T_2 pulse sequences were applied to study relaxation processes in the studied samples. Longitudinal relaxation times (T_1) were determined by using a saturation-recovery (SR) pulse sequence [65] in 50 recovery delays with a logarithmic spacing. Transverse relaxation times (T_2) were determined by employing the multipulse Carr-Purcell-Meiboom-Gill (CPMG) pulse sequence [66]. Relaxation maps were constructed by combining the pulse sequences for T_1 and T_2 determination. Four averaging scans, including phase cycling, were used. The bidimensional NMR data matrices acquired were processed with an inverse Laplace transform, which generates a T_1-T_2 correlation map [67–69].

Experimental S3. Kinetic and adsorption capacity assessment

The kinetics of adsorption were determined by measuring the sorbate concentration decay in the solution supernatant during the adsorption process. Adsorption tests were conducted in batch at room temperature (25°C), and with a constant stirring (100 rpm). Adsorption isotherms data were determined using 5 mg of dried hydrogel added to an aqueous solution (100 mL) of MO, Cu^{2+}, or PEN with concentrations ranging from 4 to 45 mg L^{-1}; 6 to 60 mg L^{-1}, and 2 to 18 mg L^{-1} respectively. The pH values for the adsorption experiments are indicated in the manuscript for each contaminant.

Adsorption tests were conducted in batch at room temperature (25 °C), and with a constant stirring (100 rpm). Adsorption isotherms data were determined using 5 mg of dried hydrogel added to an aqueous solution (100 mL) of MO, Cu^{2+}, or PEN with concentrations ranging from 4 to 45 mg L^{-1}; 6 to 60 mg L^{-1}, and 2 to 18 mg L^{-1} respectively. The concentration difference after reaching the equilibrium allows estimating the adsorption capacity of each of the studied materials at a given concentration of the pollutant. In parallel, the kinetics of adsorption were determined by measuring the sorbate concentration decay in the solution supernatant during the adsorption process. To this end, MO, Cu^{2+} and PEN solutions with 60, 30 and 12 mg L^{-1} concentrations were employed to perform the experiments. The concentration of MO, Cu^{2+}, and PEN before, during and after adsorption were determined on a Jasco 7850 UV-Vis spectrometer (Easton, Maryland, USA). MO presented a characteristic peak at 468 nm. For Cu^{2+}, the chromogenic complexing agent 1-(2-pyridylazo)-2-naphtol (PAN)[70] was used, obtaining a Cu^{2+} complex which presented a characteristic absorption peak at 548 nm. PEN presented a characteristic peak at 220 nm. For the three target pollutants studied in this work, the calibration curves were constructed. The experimental data obtained from the adsorption experiments were adjusted to kinetics and isotherm models

Experimental S4. Kinetic and Isotherm models

Adsorption kinetics

Pseudo-1st order and pseudo-2nd order equations were used as described by:[71]

$$q_t = q_{eq}(1 - e^{-k_1 . t}) \quad (1)$$

$$q_t = \frac{q_{eq}^2 . k_2 . t}{1 + (q_{eq} . k_2 . t)} \quad (2)$$

where q_t and q_{eq} are adsorption capacities at time t (h) and at equilibrium respectively (mg g^{-1}), k_1 (h^{-1}) and k_2 (g mg^{-1} h^{-1}) are the sorption rate constants for the pseudo-1st and pseudo-2nd order models, respectively. The initial sorption rate h_0 (mg g^{-1} h^{-1}) for the pseudo-2nd order kinetic model was written as: $h_0 = q_{eq}^2\ k_2$.

Considering $q_t = q_t$ at $t = t$ and $q_t = 0$ at $t = 0$, the Elovich rate equation becomes:[72]

$$q_t = \frac{1}{\beta} \ln\,(1 + (\alpha . \beta . t)) \quad (3)$$

where constant α (mg g^{-1} h^{-1}) is the initial adsorption rate and β (g mg^{-1}) is related to the extent of surface coverage and the activation energy involved in chemisorption processes.[73] This equation assumes that the active sites of the sorbent are heterogeneous in nature and therefore exhibit different activation energies for chemisorption.[72]

The modified Freundlich model was originally developed by Kuo and Lotse and is described by:[74]

$$q_t = k_F . C_0 . t^{1/m} \quad (4)$$

where k_F (L/g h) is the apparent adsorption rate constant, C_0 (mg L^{-1}) the initial sorbate concentration and m (dimensionless) is the Kuo-Lotse constant.[74] This model can describe surface diffusion-controlled processes. Particularly, it can describe kinetics controlled by intra-particle diffusion when m approaches a value of 2.[75]

Adsorption isotherms

Adsorption capacities (q_{eq}) are expressed as the moles of sorbate per mass unit of sorbent (mmol/g) and determined as follows:

$$q_{eq} = (C_0 - C_{eq})V / m \quad (5)$$

where C_0 and C_{eq} are the initial and the equilibrium sorbate concentrations of the incubation solution respectively (mg L^{-1}), V is volume of solution (L) and m is the sorbate mass (g).

Two parameters adsorption isotherms

Langmuir and Freundlich models have been widely applied to the adjustment of biosorption equilibrium data. The former assumes that a sorbate interacts homogeneously, at homogeneous sorption sites, until a monolayer is formed in the sorbent surface. On the other hand, Freundlich model have proved to describe better the adjustment of sorbents with heterogeneous adsorption sites and dissimilar interactions.[11] Langmuir and Freundlich adsorption isotherms can be expressed using equations (7) and (8) respectively:[76]

$$q_{eq} = \frac{q_m.K_a.C_{eq}}{1 + K_a.C_{eq}} \quad (7)$$

$$q_{eq} = k.C_{eq}^{n} \quad (8)$$

where K_a is the adsorption equilibrium constant (L mg^{-1}), q_m is the maximum adsorption capacity (mg g^{-1}) and k and n are arbitrary parameters. The dimension of k depends on the value of n.

Tables.

Table S1. Fitting parameters from C 1*s* XPS data.

C 1*s* PEI-EGDE								
**Band**	**Pos**	**PosSep**	**B_FWHM**	**FWHM**	**Height**	**%Gauss**	**Area**	**%Area**
1	284.8	0	1.43	1.43	8273	92	13051	67.53
2	286.0	1.25	1.43	1.43	2361	100	3597	18.61
3	286.9	2.14	1.43	1.43	1209	100	1841	9.53
4	288.8	4	1.43	1.43	549	100	837	4.33
**C 1*s* PEI-EGDE-TPP (P1T0.01)**								
**Band**	**Pos**	**PosSep**	**B_FWHM**	**FWHM**	**Height**	**%Gauss**	**Area**	**%Area**
1	284.8	0	1.6	1.6	7056	80	9968	50.28
2	286.1	1.28	1.5	1.5	4765	80	6419	38.38
3	287.2	2.37	1.5	1.5	760	80	1023	9.37
4	288.4	3.58	1.5	1.5	304	100	410	1.97
**C 1*s* PEI-EGDE-TPP (P1T0.05)**								
**Band**	**Pos**	**PosSep**	**B_FWHM**	**FWHM**	**Height**	**%Gauss**	**Area**	**%Area**
1	284.5	0	1.6	1.6	17055	80	31810	52.51
2	285.9	1.34	1.5	1.5	13887	100	24282	36.27
3	287.1	2.61	1.5	1.5	3391	100	5930	8.9
4	288.4	3.84	1.5	1.5	782	100	1249	2.32
**C 1*s* PEI-EGDE-TPP (P1T0.1)**								
**Band**	**Pos**	**PosSep**	**B_FWHM**	**FWHM**	**Height**	**%Gauss**	**Area**	**%Area**
1	284.8	0	1.27	1.27	33697	90	62850	55.94
2	286.3	1.43	1.27	1.27	27189	100	43412	36.02
3	287.5	2.69	1.27	1.27	6673	100	10654	5.74
4	288.8	4.03	1.27	1.27	1737	100	2774	2.3

Table S2. Fitting parameters from O 1*s* XPS data.

O 1*s* PEI-EGDE								
Band	Pos	PosSep	B_FWHM	FWHM	Height	%Gauss	Area	%Area
1	532.3	0	1.6	1.6	5288	90	9418	69.98
2	533.4	1.18	1.5	1.5	2311	80	4041	30.02
O 1*s* PEI-EGDE-TPP (P1T0.01)								
Band	Pos	PosSep	B_FWHM	FWHM	Height	%Gauss	Area	%Area
1	531.0	0	1.68	1.68	975	76	1938	14.39
2	532.4	1.42	1.54	1.54	6139	100	10031	74.48
3	533.6	2.6	1.5	1.5	872	84	1499	11.13
O 1*s* PEI-EGDE-TPP (P1T 0.05)								
Band	Pos	PosSep	B_FWHM	FWHM	Height	%Gauss	Area	%Area
1	530.8	0	1.65	1.65	3684	90	6778	16.75
2	532.2	1.43	1.55	1.55	13473	80	24344	60.14
3	533.2	2.44	1.55	1.55	4977	70	9355	23.11
O 1*s* PEI-EGDE-TPP (P1T 0.1)								
Band	Pos	PosSep	B_FWHM	FWHM	Height	%Gauss	Area	%Area
1	530.7	0	1.65	1.65	9260	90	17037	20.61
2	532.1	1.42	1.55	1.55	29079	89	50425	61
3	533.1	2.47	1.55	1.55	8406	79	15203	18.39

Table S3. Fitting parameters from N 1*s* XPS data.

N 1*s* PEI-EGDE								
Band	Pos	PosSep	B_FWHM	FWHM	Height	%Gauss	Area	%Area
1	399.7	0	2	2	110	80	257	69.82
2	401.8	2.09	2	2	48	80	111	30.18
N 1*s* PEI-EGDE-TPP (P1T0.01)								
Band	Pos	PosSep	B_FWHM	FWHM	Height	%Gauss	Area	%Area
1	399.6	0	1.65	1.65	597	100	1046	62.56
2	401.7	2.12	2.2	2.2	267	100	626	37.44
N 1*s* PEI-EGDE-TPP (P1T0.05)								
Band	Pos	PosSep	B_FWHM	FWHM	Height	%Gauss	Area	%Area
1	399.6	0	2.08	2.08	1955	80	4735	59.02
2	401.6	2.04	1.92	1.92	1466	80	3288	40.98
N 1*s* PEI-EGDE-TPP (P1T0.1)								
Band	Pos	PosSep	B_FWHM	FWHM	Height	%Gauss	Area	%Area
1	399.3	0	2.08	2.08	4830	80	11717	63.78
2	401.1	1.84	1.92	1.92	3262	100	6654	36.22

Table S4. Fitting parameters from P 2*sp* XPS data.

P 2*p* PEI-EGDE-TPP (P1T0.01)								
Band	Pos	PosSep	B_FWHM	FWHM	Height	%Gauss	Area	%Area
1	133.5	0	1.56	1.56	121	94	206	66.67
2	134.3	0.84	1.56	1.56	59	90	103	33.33
P 2*p* PEI-EGDE-TPP (P1T0.05)								
Band	Pos	PosSep	B_FWHM	FWHM	Height	%Gauss	Area	%Area
1	133.2	0	2.05	2.05	168	80	402	66.67
2	134.0	0.84	2.05	2.05	84	80	201	33.33
P 2*p* PEI-EGDE-TPP (P1T0.01)								
Band	Pos	PosSep	B_FWHM	FWHM	Height	%Gauss	Area	%Area
1	133.2	0	1.85	1.85	1198	98	2381	66.67
2	134.1	0.84	1.85	1.85	551	80	1190	33.33

Figures.

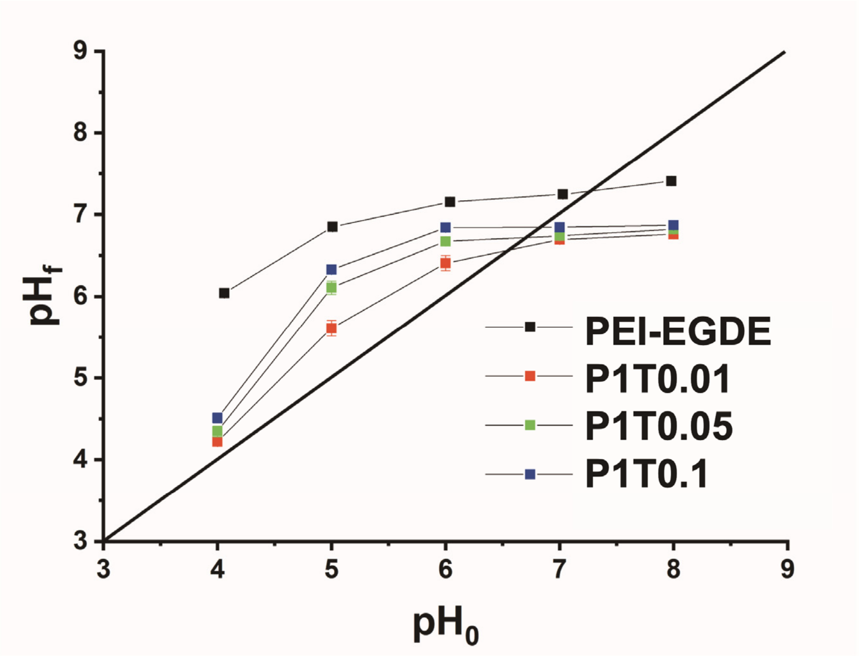

Figure S1. pH_{pzc} for the PEI-EGDE, P1T0.01, P1T0.05 and P1T0.1 hydrogels.

References

61. Sun, H.; Zhan, J.; Chen, L.; Zhao, Y. Preparation of CTS/PAMAM/SA/Ca2+ Hydrogel and Its Adsorption Performance for Heavy Metal Ions. *Appl. Surf. Sci.* **2023**, *607*, 155135. https://doi.org/10.1016/j.apsusc.2022.155135.
62. Lopez-Ramon, M.V.; Stoeckli, F.; Moreno-Castilla, C.; Carrasco-Marin, F. On the Characterization of Acidic and Basic Surface Sites on Carbons by Various Techniques. *Carbon. N. Y.* **1999**, *37*, 1215–1221. https://doi.org/10.1016/S0008-6223(98)00317-0.
63. Brownstein, K.R.; Tarr, C.E. Importance of Classical Diffusion in NMR Studies of Water in Biological Cells. *Phys. Rev. A* **1979**, *19*, 2446–2453. https://doi.org/10.1103/PhysRevA.19.2446.
64. Grebenkov, D.S. Multiexponential Attenuation of the CPMG Spin Echoes Due to a Geometrical Confinement. *J. Magn. Reson.* **2006**, *180*, 118–126. https://doi.org/10.1016/j.jmr.2006.01.014.
65. Markley, J.L.; Horsley, W.J.; Klein, M.P. Spin-Lattice Relaxation Measurements in Slowly Relaxing Complex Spectra. *J. Chem. Phys.* **1971**, *55*, 3169–3177. https://doi.org/10.1063/1.1676626.
66. Carr, H.Y.; Purcell, E.M. Effects of Diffusion on Free Precession in Nuclear Magnetic Resonance Experiments. *Phys. Rev.* **1954**, *94*, 630–638. https://doi.org/10.1103/physrev.94.630.
67. Venkataramanan, L.; Song, Y.-Q.; Hürlimann, M.D. Solving Fredholm Integrals of the First Kind with Tensor Product Structure in 2 and 2.5 Dimensions. *IEEE Trans. Signal Process* **2002**, *50*, 1017–1026.
68. Mitchell, J.; Chandrasekera, T.C.; Gladden, L.F. Numerical Estimation of Relaxation and Diffusion Distributions in Two Dimensions. *Prog. Nucl. Magn. Reson. Spectrosc.* **2012**, *62*, 34–50. https://doi.org/10.1016/j.pnmrs.2011.07.002.
69. Teal, P.D.; Eccles, C. Adaptive Truncation of Matrix Decompositions and Efficient Estimation of NMR Relaxation Distributions. *Inverse Probl.* **2015**, *31*, 045010. https://doi.org/10.1088/0266-5611/31/4/045010.
70. Cheng, K.L.; Bray, R.H. 1-(2-Pyridylazo)-2-Naphthol as a Possible Analytical Reagent. *Anal. Chem.* **1955**, *27*, 782–785. https://doi.org/10.1021/ac60101a024.
71. Ho, Y.S.; McKay, G. The Kinetics of Sorption of Divalent Metal Ions onto Sphagnum Moss Peat. *Water Res.* **2000**, *34*, 735–742. https://doi.org/10.1016/S0043-1354(99)00232-8.
72. Cheung, C.W.; Porter, J.F.; Mckay, G. Sorption Kinetic Analysis for the Removal of Cadmium Ions from Effluents Using Bone Char. *Water Res.* **2001**, *35*, 605–612. https://doi.org/10.1016/S0043-1354(00)00306-7.
73. Teng, H.; Hsieh, C.T. Activation Energy for Oxygen Chemisorption on Carbon at Low Temperatures. *Ind. Eng. Chem. Res.* **1999**, *38*, 292–297. https://doi.org/10.1021/ie980107j.
74. Kuo, S.; Lotse, E.G. Kinetics of Phosphate Adsorption and Desorption by Hematite and Gibbsite. *Soil. Sci.* **1973**, *116*, 400–406.
75. Anirudhan, T.S.; Rejeena, S.R.; Tharun, A.R. Preparation, Characterization and Adsorption Behavior of Tannin-Modified Poly(Glycidylmethacrylate)-Grafted Zirconium Oxide-Densified Cellulose for the Selective Separation of Bovine Serum Albumin. *Colloids Surf. B Biointerfaces* **2012**, *93*, 49–58. https://doi.org/10.1016/j.colsurfb.2011.12.010.
76. Suen, S.-Y. A Comparison of Isotherm and Kinetic Models for Binary-Solute Adsorption to Affinity Membranes. *J. Chem. Technol. Biotechnol.* **1996**, *65*, 249–257. https://doi.org/10.1002/(sici)1097-4660(199603)65:3<249::aid-jctb411>3.0.co;2-m.
